# Loss of the Cochlear Amplifier Prestin Reduces Temporal Processing Efficacy in the Central Auditory System

**DOI:** 10.3389/fncel.2018.00291

**Published:** 2018-09-21

**Authors:** Joseph P. Walton, Adam C. Dziorny, Olga N. Vasilyeva, Anne E. Luebke

**Affiliations:** ^1^Department of Communication Sciences and Disorders, University of South Florida, Tampa, FL, United States; ^2^Department of Chemical and Biomedical Engineering, University of South Florida, Tampa, FL, United States; ^3^Global Center for Hearing and Speech Research, University of South Florida, Tampa, FL, United States; ^4^Department of Biomedical Engineering, University of Rochester, Rochester, NY, United States; ^5^Department of Neuroscience, The Ernest J. Del Monte Institute for Neuroscience, University of Rochester Medical Center, Rochester, NY, United States

**Keywords:** prestin, cochlear amplifier, central auditory processing, ABR, inferior colliculus, gap detection, tuning, frequency

## Abstract

Active mechanical amplification of sound occurs in cochlear outer hair cells (OHCs) that change their length with oscillations of their membrane potential. Such length changes are the proposed cellular source of the cochlear amplifier, and prestin is the motor protein responsible for OHC electromotility. Previous findings have shown that mice lacking prestin displayed a loss of OHC electromotility, subsequent loss of distortion-product otoacoustic emissions, and a 40–60 dB increase in hearing thresholds. In this study we were interested in studying the functional consequences of the complete loss of cochlear amplification on neural coding of frequency selectivity, tuning, and temporal processing in the auditory midbrain. We recorded near-field auditory evoked potentials and multi-unit activity from the inferior colliculus (IC) of prestin (−/−) null and prestin (+/+) wild-type control mice and determined frequency response areas (FRAs), tuning sharpness, and gap detection to tone bursts and silent gaps embedded in broadband noise. We were interested in determining if the moderate to severe sensorineural hearing loss associated with the loss of motor protein prestin would also impair auditory midbrain temporal-processing measures, or if compensatory mechanisms within the brainstem could compensate for the loss of prestin. In prestin knockout mice we observed that there are severe impairments in midbrain tuning, thresholds, excitatory drive, and gap detection suggesting that brainstem and midbrain processing could not overcome the auditory processing deficits afforded by the loss of OHC electromotility mediated by the prestin protein.

## Introduction

Outer hair cells (OHCs) function as “cochlear amplifiers," and are responsible for generating force leading to the expansive range of auditory sensitivity and frequency selectivity (Fettiplace and Hackney, 2006). In mammals, active mechanical amplification of sound can occur in cochlear OHCs which change their length with oscillations of their membrane potential. Such length changes are the proposed cellular source of the cochlear amplifier, and prestin is the motor protein responsible for OHC electromotility, which improves auditory sensitivity by 100-fold, or approximately 40 dB (Liberman et al., 2002).

In studies of prestin KO mice, significant alteration in frequency tuning was found and auditory nerve fiber characteristic frequencies shifted to lower frequencies, with the expected loss in auditory sensitivity (Santos-Sacchi, 2008). In these KO mice, the OHCs were approximately 60% shorter in length and had a reduced stiffness compared to normal mouse hair cells, indicating abnormal cochlear micromechanics. This prompted the study of a knockin mouse with both diminished prestin function and normal length and stiffness hair cells. Recordings of the compound action potential demonstrated that these mice had similar auditory function to the original KO mice, confirming the importance of prestin for cochlear amplification (Dallos et al., 2008).

Prestin may also be implicated in age-related hearing loss (ARHL) as a study by Chen et al. (2009) found that OHCs from aged rats were present, though dysfunctional, resulting in reduced otoacoustic emissions. Molecular assays showed that expression of prestin was decreased in aged OHCs, which was believed to be a major cause of this age-related loss in function and cochlear sensitivity (Chen et al., 2009). Non-syndromic deafness in humans also arises from mutations in the prestin gene, specifically from a splicing junction mutation resulting in moderate to severe congenital hearing loss (Geleoc and Holt, 2003; Liu et al., 2003).

In this study we used a prestin KO mouse (Liberman et al., 2002) to study the functional consequences of the complete loss of cochlear amplification on frequency selectivity and temporal processing in the auditory midbrain. We recorded both near-field auditory evoked potentials (NFAEPs) and multi-unit activity to tone bursts and silent gaps embedded in broadband noise from the inferior colliculus (IC) of prestin (–/–) KO and prestin (+/+) wild-type, control mice. We were interested in determining if the severe sensorineural hearing loss associated with the loss of prestin would also impair auditory midbrain temporal-processing.

## Materials and Methods

### Animals

Prestin-null heterozygote (+/−) breeder pairs were generously provided by Dr. Jian Zuo (St. Jude Research Hospital, Memphis, TN, United States) which were generated originally from ES cells from the 129 SvEv strain, and have been backcrossed to the C57Bl/6J (C57) strain for >25 generations (Liberman et al., 2002). The Prestin- line was maintained and carried as heterozygotes (+/−). The mice used for this study were bred as homozygotes from (+/+ or −/−) offspring of prestin heterozygous pairings, using at least three separate litters of (+/+) and (−/−) mice. Mice were maintained on a 12 h light/dark cycle with ad lib access to water and food pellets.

As prestin KO mice demonstrate a loss of OHCs at high-frequencies (>30 kHz), followed by a rapid progression of OHC loss, as early as postnatal day (P) P35 (Wu et al., 2004), we chose to test all experimental animals at after 32 days of age. Auditory brainstem responses (ABRs) and near-field IC recordings were completed on 12 wild-type and 14 prestin KO mice. The mean age of wild-type mice was 28.5 (±1.0) days, and prestin KO mice was 28 (±1.8) days. Extracellular multi-unit recordings from the IC were completed on 4 wild-type (+/+) mice (ages P29–P36), and 6 prestin KO (−/−) mice (ages P28–P32).

The University of Rochester’s Committee on Animal Resources in accordance with the National Institutes of Health Guidelines approved all procedures for the Care and Use of Animals.

### Auditory Brainstem Response Audiometry

Animals were anesthetized with ketamine/xylazine (100/10 mg/kg, intraperitoneal) and body temperature was maintained throughout the recording with a heating pad. ABRs were recorded from needle electrodes placed below the skin at the vertex and pinna, with a ground electrode at the contralateral pinna. Stimuli were generated using BioSig Software [Tucker Davis Technologies (TDT), Alachua, FL, United States], and consisted of 5 ms tone and noise bursts (0.5 ms rise-fall with a cos^2^ envelope) delivered at 25/s. Each presentation consisted of >150 averages, with two presentations per level. Responses were amplified (10,000×), filtered (300–3,000 Hz), and sampled at 24 kHz using a TDT System II A–D converter. Stimuli were presented in 5 dB increments from 80 dB SPL to 15 dB below the visual threshold. Visible threshold was defined as the lowest level at which discernable waveforms were present in the replicates. Peaks were automatically selected and verified by eye using custom software that measured peak latencies and amplitudes.

### Surgical Preparation

The anesthesia, surgical preparation, and tranquilization procedures are described in detail elsewhere (Barsz et al., 2007). Briefly, at least 24 h after ABR assessment and 24 h prior to near-field recording and unit recording, mice were anesthetized and the cranium was exposed so a small metal tube could be glued to the skull. A tungsten indifferent electrode was implanted in the skull to contact the dura.

For IC and near-field recording, mice were briefly anesthetized with isofluorane (Isofane^®^) and tranquilized with chlorprothixene (Taractin^®^2.5 g/kg). Mice were placed in a molded plastic support, inside a heated (28–31°C) chamber (ICS) lined with sound-absorbing foam (Sonex^®^). The head was immobilized by connecting the anchored metal tube to a custom stereotaxic frame (Newport Klinger, Irvine, CA, United Sates). The IC was located stereotaxically and a small hole was drilled through the skull. The opening was kept moist with saline throughout the recording session, which typically lasted 6–8 h. The mouse was continuously monitored and if any signs of discomfort were noted it was removed from the apparatus.

Initially the IC was mapped in order to confirm the electrode was in the central nucleus. Near-field neural responses were recorded using a single tungsten electrode (20–40 μm tip diameter, 150–400 kΩ), lowered into the IC with a micropositioner (Newport-Klinge Micropositioner 750). Neural activity was recorded between the recording and indifferent electrodes, amplified 10,000×, band-passed filtered (10–3 kHz), and digitally sampled at 40 kHz using a TDT RX5. Responses to tone stimuli were recorded on the surface and every 100 μm as the electrode was being advanced. NFAEPs were recorded at two locations, dorsal recordings were made 500 μm from the surface, and ventral recordings were made 1,500 μm. Only one electrode penetration was performed on each animal. Near-field recording sessions typically lasted 4 h.

Multi-channel recordings were made using a vertically oriented 16-channel electrode (part # a1 × 16–3 mm 100–177, NeuroNexus Technologies, Ann Arbor, MI, United States) with electrode pads spaced 100 μm apart, each having an impedance of 2 MΩ. Electrodes were lowered into the IC using a micropositioner (Newport-Klinger PMC 100) while presenting search stimuli until driven activity disappeared from the most ventral electrode pad (typically 1.6–2.0 mm total). Electrode positions were verified to be in IC by histological analysis (data not shown). The output of the electrode was led to a low-noise preamplifier (TDT RA16), filtered (300–3 kHz), amplified and sampled at 25 kHz. Neural events were visualized in real-time using the OpenEx software platform (TDT) and a custom MatLab graphical interface. A voltage discriminator was automatically set at 4:1 SNR on each channel, and events were time-stamped and stored for off-line analysis. Excitatory receptive fields were quantified from on 233 units (65%) of wild-type mice and 316 (80%) of prestin KO mice; responses from the remaining MUs were not driven (i.e., the response to tones was below 2 spikes/s).

### Near-Field Stimulus Protocol

Near-field stimuli details are as described previously (Allen et al., 2003). Briefly, all stimuli were generated and presented using TDT hardware and software. All stimuli were attenuated (TDT PA4), amplified (Kenwood 620), and broadcast from a Panasonic Model 203 leaf tweeter (which varies <6 dB from 2 to 50 kHz). The speaker was positioned 20 cm from the mouse’s head, 30° from the midline on the side contralateral to the recording and calibrated using a B&K 2,610 amplifier and a ¼″ microphone placed at the location of the pinna.

Tone bursts consisted of brief 5 ms tone bursts with 0.5 ms linear rise-fall, presented at 3, 6, 12, 24, and 36 kHz from 80 to 0 dB in 5 dB increments. Rate-level stimuli consisted of broad-band noise bursts which were 50 ms in duration (1 ms nominal cos^2^ rise-fall time, 100 kHz sampling rate). Pairs of noise bursts separated by silent gaps of with durations between 1.0 and 64 ms, in addition to a no-gap condition, and were presented using an interstimulus interval of 200 ms and used to evaluate neural gap encoding.

### Multi-Unit Stimulus Protocol

The methods used to acquire the multi-unit data has been described previously (Leong et al., 2009). Briefly, all stimuli were generated digitally using a System 3 digital signal processor (RX6) and presented using TDT software and hardware. The signals were presented through a speaker (TDT ES1) positioned 22.5 cm from the pinna at 60° from the midline on the side contralateral to the recording. The system was calibrated using a B&K 2610 amplifier and a ¼″ microphone at the location of the pinna.

Stimulus presentation was controlled through custom MatLab software (The MathWorks, Inc., Natick, MA, United Sates) using the TDT OpenEx interface. Wideband, 50 ms duration, noise bursts were used as search signals and presented at 70 dB SPL at a rate of 5/s. Frequency response areas (FRAs) were generated using 25 ms tone bursts having frequencies from 2 to 64 kHz (500 Hz steps) and presented from 0 to 85 dB (5 dB steps). Five repetitions of each of the 2,125 frequency-level combinations were presented; with all pairs being presented in random order at a rate of 10/s. Rate-level functions (RLFs) were measured with 100 ms broadband (1–100 kHz) noise bursts from 0 to 80 dB in 5 dB steps. Gap stimuli consisted of two broadband (1–100 kHz) noise bursts (NB1, 100 ms and NB2, 50 ms in duration) with gaps of varied duration (0, 1, 2, 4, 8, 16, 32, 64, or 96 ms) embedded following the first noise burst (NB1). Noise bursts were presented at 80, 70, and 60 dB SPL for wild-type animals, and at 80 dB SPL only for prestin KO animals. Background noise was added to gap stimuli at -6 dB signal-to-noise ratio (SNR) in order to test the effects of background noise on gap encoding.

### Spike Sorting

Spike waveforms were processed offline using custom Matlab routines. The set of voltage-time waveforms were sorted using a Bayesian unsupervised algorithm (AutoClass 3.3.5, NASA Ames Laboratory, Moffet Field, CA, United Sates) that seeks a maximum posterior probability classification. To better discriminate signal from noise, we estimated the variance of the background noise using the set of five points recorded immediately prior to the threshold-crossing event. Events were screened prior to classification, such that only time points with voltage values greater than twice the noise floor were submitted to AutoClass. This greatly reduced the over-classification of event data. The classes were then reviewed by two independent experimenters and characterized as single-unit, multi-unit or noise, based on characteristic waveform features. Single units were identified based on minimal spike variance, high (>10:1) SNR, and biphasic profile. When spikes could not be ascribed to a single neuron, they were classified as a multiple unit cluster. As in previous studies (Mickey and Middlebrooks, 2003; Stecker et al., 2003; Harrington et al., 2008), both single-units and multiple-unit clusters are referred to as “units.” Of the 749 units recorded in this study, 104 (14%) were single-units. Events classified as noise were subsequently discarded.

### Data Analysis

Frequency Response areas were displayed in Matlab and analyzed with a multi-step procedure using custom software. Quantification was only completed on units with driven response to tones >25 spikes/s. First, the spontaneous rate was estimated as the mean response to low-frequency, low-level stimuli (Sutter and Schreiner, 1991; Schreiner and Sutter, 1992). The mean spike rate was computed from the lower-left corner of the FRA (2–4.5 kHz, 0–15 dB SPL). No units exhibited driven activity within this region. Second, the maximum rate of the FRA was found. Third, each FRA was smoothed using a 3 × 3 moving average. The edges of the response were defined as activity greater than background rate plus 15% of the maximum rate. The best frequency (BF) was identified as the frequency with the lowest intensity of driven activity (threshold). *Q*-values were determined automatically at each 10 dB level above threshold (up to 60 dB above threshold) using the following formula: Q_XdB_ = (Best Frequency)/(Bandwidth at X dB above threshold). *Q*-values were not calculated for any level that exceeded the maximum level of the FRA (85 dB).

We examined changes in individual unit excitatory drive using tone and noise RLFs. Tone functions were derived from the FRA at the BF of each unit. The number of spikes was counted in response to the BF tone at each level from 0 to 80 dB in 5 dB increments. Noise functions were generated by counting the number of spikes in the first 25 ms of a 100 ms broadband noise burst, presented from 0 to 80 dB in 5 dB increments. Several measurements were derived from these functions, including maximum rate, the 10, 50, and 90% of the maximum rate, and the intensity levels that elicited those rates.

Neural responses to gaps in noise were analyzed using a previously described method (Barsz et al., 2000, 2002; Wilson and Walton, 2002; see **Figure 1** in Walton et al., 2007). Briefly, units were excluded from gap analysis if they were not driven (>2 spikes/s) by noise bursts at the given stimulus level. Composite post-stimulus time histograms (PSTHs) were generated for each unit by summing individual PSTHs. Two comparison windows were used, as gap encoding for tonic units typically occurs during the gap, while for phasic units it occurs after NB2 onset. First, histograms were summed with respect to NB1 (Walton et al., 2007). The quiet window (QW) was defined as beginning at the offset of the NB1 response and continuing for the duration of the gap. The count of spikes in this window was compared to the count of spikes in the same time period in the control condition (0 ms). Second, histograms were summed with respect to NB2 by shifting each histogram back in time (i.e., to the left) by that histogram’s gap duration and then summing over the shifted PSTHs. The onset of the response to NB2 was determined automatically at the point equaling 10% of the maximum response to NB2. A second window was defined beginning at the NB2 onset and continuing for 25 ms, and again the count of spikes in this window was compared to the count of spikes in the same time period in the control condition (0 ms). The Wilcoxin signed-rank test was used for these comparisons. The minimum gap threshold (MGT) was defined as the gap duration having a *p* < 0.05 using either the quiet or driven window, provided the next-largest gap duration was also significant using that window (Wilson and Walton, 2002).

**FIGURE 1:**
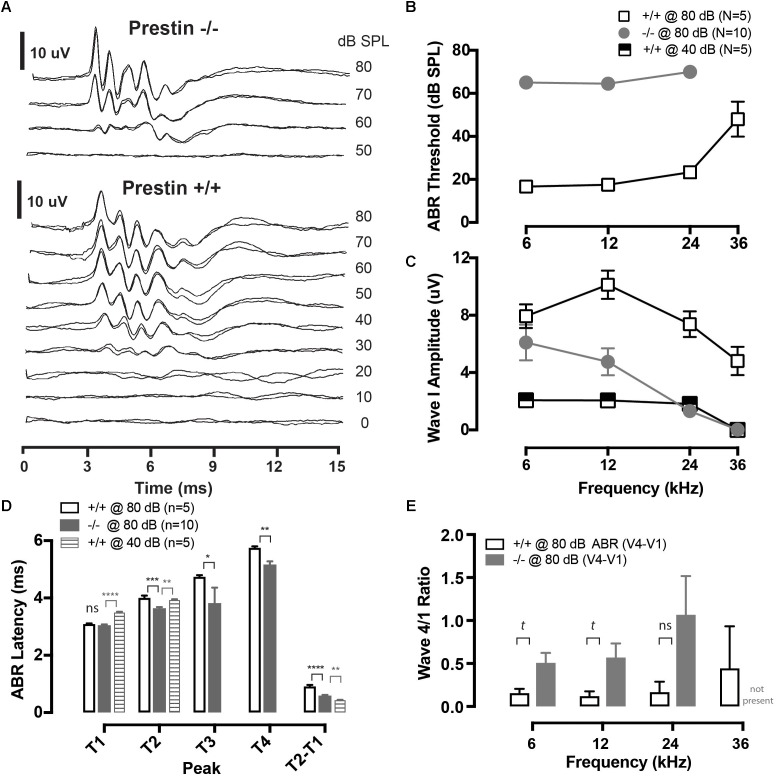
Cochlear function in prestin KO (–/–) mice. **(A)** Representative auditory brainstem response (ABR) waveforms from wild-type (+/+) and prestin KO (–/–) mice plotted as a function of intensity for a 12 kHz tone burst. Each intensity was replicated. **(B)** ABR thresholds are elevated >40 dB across all frequencies in prestin KO (–/–) mice compared to wild-type (+/+) controls. **(C)** ABR peak 1 amplitude is also decreased in prestin KO (–/–) mice yet either greater than or equal to equal sensation levels to controls at 40 dB. **(D)** ABR latencies (12 kHz; 80 dB) in prestin KO (–/–) mice (gray bars) are decreased for P2, P3, and P4 compared to controls @ 80 dB (open bars) and are P2 is decreased at equal sensation levels when compared to controls @ 40 dB (gray striped bars), but are not significantly different from P1. Interpeak latencies of P2-P1 @ 80 dB are also significantly prolonged. **(E)** There is no significant difference between ABR peak 4 divided by peak 1 amplitudes (P4/P1), yet there was a trend (*t*) for peak P4/P1 to be greater in the prestin KO animals, perhaps reflecting increased central compensation (*p* = 0.0553). Plots are mean ± SEM. (*^t^p* < 0.1, ^∗^*p* < 0.05; ^∗∗^*p* < 0.01; ^∗∗∗^*P* < 0.001, ^∗∗∗∗^*p* < 0.0001).

Recovery functions are plotted as NB2/NB1 × 100 spike count (in a 25 ms window following NB2 onset) versus gap duration. Recovery function slopes are calculated on a unit-by-unit basis by fitting a line to the plot of NB2 counts versus gap duration, for all gap durations greater than or equal to the MGT.

Near-field auditory evoked potentials were analyzed using a fully automated algorithm designed in LabView (National Instruments). This analysis has been described in detail previously (Allen et al., 2003, 2008). Briefly, analysis was performed by calculating the root mean square (RMS) voltage over a duration of 30 ms. Spike counts for computing RLFs were calculated from 25 ms time windows at the onset of either the tone or noise stimulus. Analysis of gap-in-noise stimuli required windows at the onset of both noise bursts (NB1 and NB2). The ratio of NB2 onset to NB1 onset in each NFAEP was calculated. Gap recovery functions using this metric demonstrate the fraction of recovery for each gap duration. Fitting these functions to a sigmoid and identifying the duration in ms of a 10% rise from baseline to saturation determined gap thresholds.

### Statistics

The summary data was graphed in the form of mean and standard error of the mean using Graph Pad Prism software (version 5), and all statistical analyses were computed with SPSS. The Student’s *t*-test was used to examine differences between two groups, while univariate analysis of variance was used to compare differences among groups of data. Either Tukey or Bonferroni *post hoc* tests were used for multiple comparisons. When necessary, we also employed non-parametric statistical testing using Mann–Whitney and Kruskal–Wallis using a *p*-value of <0.05 as significant, and a *p*-value <0.1 considered a trend.

## Results

Auditory brainstem responses were recorded from 5 wild-type mice and 10 prestin KO mice. At suprathreshold levels (70 and 80 dB SPL) the ABR morphology is observed to be similar between prestin KO and WT mice, as is shown in **Figure 1A**. ABR thresholds were increased by approximately 40 dB at 6, 12, and 24 kHz as compared to WT mice, with no detectable response at 36 kHz in prestin KO mice (**Figure 1B**). These threshold differences are similar to those reported in previous studies of the prestin KO mouse (Liberman et al., 2002; Cheatham et al., 2004), as are peak 1 amplitude differences between wild-type and prestin KO mice (shown in **Figure 1C**).

Peak latencies for P1–P4 were measured from ABR waveforms in response to 80 dB SPL noise bursts (**Figure 1D**). Peak 2, P3, and P4 latencies were significantly shorter in prestin KO mice, while P1 latencies were unchanged when compared to control wild-type mice. Moreover, peak latencies for P2 were also shorter in the prestin KO mice when compared with equal sensation levels (40 dB) in the wild-type mice (**Figure 1D**). Interpeak latencies (P2-P1) were also computed and shown in the right bars of **Figure 1D** to comparing both wild-type at 80 dB to prestin KO at 80 dB and prestin KO at 80 dB to control mice at 40 dB. There was a trend for P4/P1 to be greater at 6 and 12 kHz in prestin KO mice (Kruskal–Wallis, *p* = 0.553). Taken together the ABR peak latency measures demonstrate an increase in speed of action potential propagation velocity between P1 and P2 of the ABR. Indicating increased speed of conduction between the auditory nerve (P1) and cochlear nucleus (P2) (Willott, 2001). While there were latency differences between wild-type and prestin KO mice there was no significant difference in amplitude between measured ABR peak 4 amplitude divided by peak 1 amplitude (4/1 ratio) as shown in **Figure 1E**, most likely due to increased variances in this amplitude ratio observed in prestin KO mice.

### Auditory Midbrain Frequency Selectivity and Tuning

**FIGURE 2:**
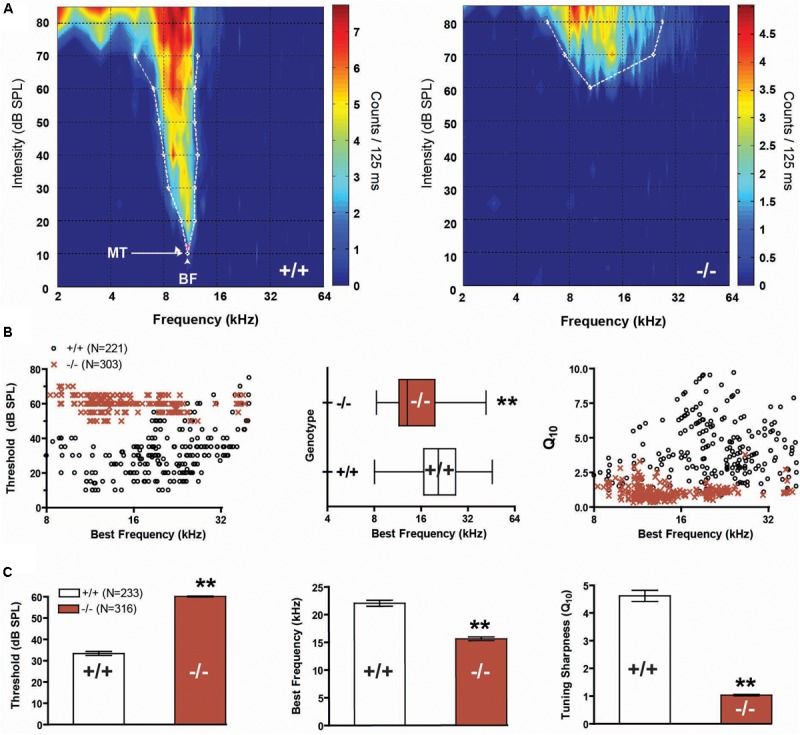
Frequency representation and tuning sharpness in the auditory midbrain. **(A)** Representative frequency response maps from wild-type (+/+) and prestin KO (–/–) mice. **(B)** Units from prestin KO (–/–) mice (dark red) display a BF shift (scatter-plot, *left*, and box-whisker, *middle*) compared to wild-type (+/+) controls. BF distributions comparing null and wild-type units were significantly different (2-sample KS test, *p* < 0.001). Tuning sharpness (Q10) was decreased in prestin KO (–/–) mice compared to wild-type (+/+) mice (*right*). **(C)** prestin KO (–/–) mice demonstrated a significant threshold increase of 30 dB (*left*), a reduction in units with high-frequency BF (*middle*), and decreased tuning sharpness (*right*) when compared to wild-type (+/+) controls. Plots are mean ± SEM.

We recorded responses from a total of 356 multi-units (MU) in wild-type mice, and 393 MUs in prestin KO mice. Qualitatively, FRAs from wild-type mice demonstrated lower thresholds and sharper tuning than responses from prestin KO mice (**Figure 2A**). In addition, many receptive fields from −/− mice, such as shown in the example, were weakly driven with sparse sound evoked activity in the upper frequencies. The BF of units in prestin KO mice were skewed toward lower frequencies, with few units responsive to 85 dB stimuli at frequencies above 24 kHz, whereas wild-type mice show a broad BF distribution across the stimulus frequency range (**Figure 2B**, *left*). Comparisons of the 2 distributions of BFs indicate that they are significantly different (2-sample Kolmogorov–Smirnov test, *p* < 0.001, **Figure 2B**, *middle*). We examined tuning sharpness (as measured by Q_10_) versus BF and found that tuning sharpness decreased or receptive fields became broader across all frequencies in prestin KO mice compared to wild-type mice (**Figure 2B**, *right*). In addition, MU minimum thresholds from prestin KO mice were increased (−/−: 60.1 dB SPL, +/+: 33.4 dB SPL, *p* < 0.001) while BFs (−/−: 15.6 kHz, +/+: 22.1 kHz, *p* < 0.001) and tuning sharpness (Q_10_) values were decreased (−/−: 1.0, +/+: 4.6, *p* < 0.001) when compared to wild-type controls (**Figure 2C**). These changes in threshold and tuning mirror previous studies of IC function in the setting of OHC loss (Sterbing and Schrott-Fischer, 2002).

### Excitatory Drive

**FIGURE 3:**
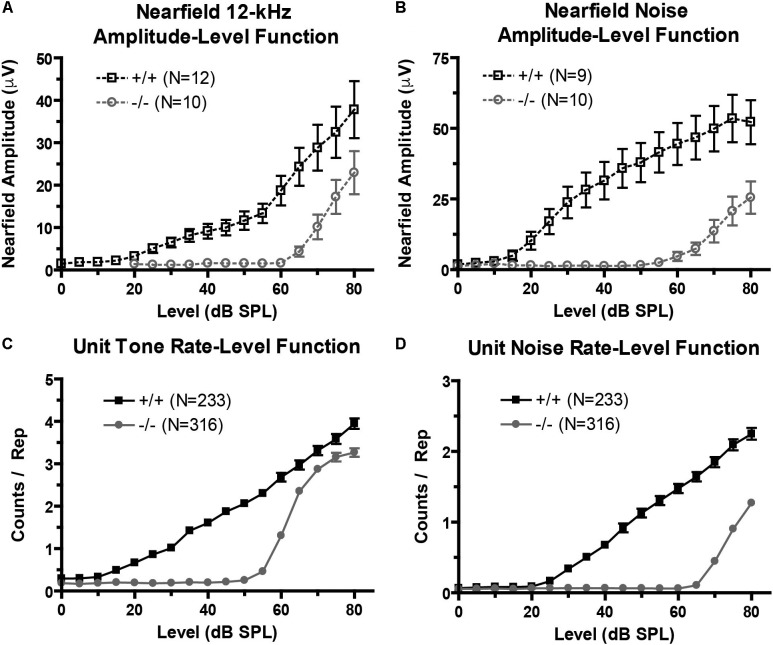
Excitatory drive is reduced in the prestin KO (–/–) mouse. **(A,B)** Near-field amplitude-level functions in response to 12 kHz tone **(A)** or broadband noise **(B)** stimuli show decreased excitatory activity in prestin KO (–/–) mice compared to wild-type (+/+) controls. **(C,D)** Unit tone **(C)** and noise **(D)** rate-level functions match near-field amplitude-level functions and confirm decreased excitatory activity. Plots are mean ± SEM.

We recorded near-field auditory evoked-potentials (NFAEPs) from 12 wild-type mice and 10 prestin KO mice. NFAEP amplitude versus stimulus level was plotted in response to a 12 kHz tone (**Figure 3A**), and broadband noise (**Figure 3B**). These functions show decreased excitatory drive from population responses to both tones and noise in the prestin KO mice compared to control mice. With both tone and noise stimuli, the NFAEP amplitude at the maximum stimulus level (80 dB) was significantly decreased in prestin KO mice (tone: 22.9 μV; noise: 25.5 μV) compared to wild-type controls (tone: 37.8 μV, noise: 52.2 μV). Multiunit tone and noise RLFs were computed on all units with FRAs (233 units from wild-type mice, 316 units from prestin KO mice). RLFs were computed from each units’ BF and indicated decreased driven responses to both tones (**Figure 3C**) and noise (**Figure 3D**) in the prestin KO mice compared to wild-type mice across all intensities. The counts per repetition at 80 dB in response to a BF tone and noise are significantly decreased in prestin KO mice (BF: 3.3; noise: 1.3) compared to wild-type controls (BF: 3.9, noise: 2.2). This agreement between population responses (NFAEPs) and unit responses demonstrates that IC drive is decreased in prestin KO mice.

### Temporal Coding

**FIGURE 4:**
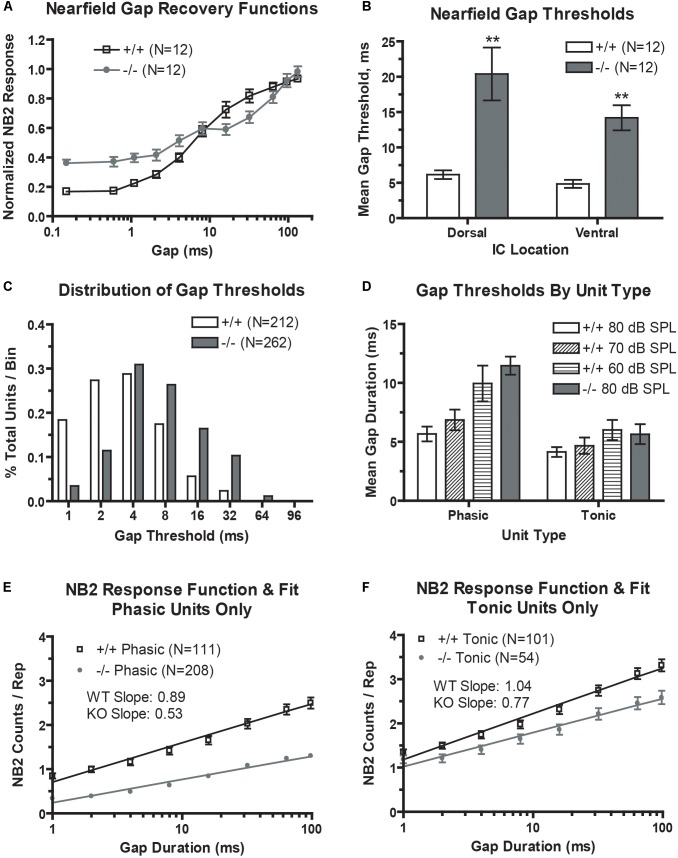
Gap encoding is impaired in the prestin KO (–/–) mouse. **(A)** prestin KO (–/–) mice demonstrate decreased dynamic range and decreased recovery slopes in near-field recover functions. **(B)** Mean minimal gap thresholds (MGT) measured from near-field responses are significantly elevated in both the dorsal and ventral IC of prestin KO (–/–) compared to wild-type (+/+) mice. **(C)** Units from prestin KO (–/–) mice show increased mean MGT as evidenced by a rightward shift in MGTs in the frequency distribution. **(D)** Mean MGTs from phasic units show a dependence on carrier level with MGT increasing with decreases in carrier intensity, while tonic units do not exhibit such dependence. **(E,F)** Both phasic **(E)** and tonic **(F)** units from the prestin KO (–/–) mouse exhibit abnormal prolonged recovery functions for the 80 dB carrier. Plots are mean ± SEM with linear fits.

We first looked at population temporal coding using NFAEPs of gap-embedded stimuli. Gap recovery functions from dorsal (low frequency) region are shown, with the NB1/NB2 amplitude plotted as a function of gap duration (**Figure 4A**). A value of 1.0 would denote complete recovery of the NB2 amplitude. The recovery function from wild-type mice demonstrates the typical sigmoidal shape with near complete recovery at a gap duration of 96 ms, matching those in previous studies (see Figure 6, Allen et al., 2003). The recovery function from prestin KO mice, however, has a smaller dynamic range (minimum to maximum driven response), a shallower slope, and no clear saturation point. Neuronal population minimal gap thresholds (**Figure 4B**) were determined from the 10% point of the recovery function. Both dorsal and ventral MGTs computed from the 10% point were significantly elevated in prestin KO mice (dorsal: 20.4 ms; ventral: 14.2 ms) compared to wild-type mice (dorsal: 6.2 ms; ventral: 4.9 ms).

We then examined neural correlates of gap-in-noise detection using an identical stimulus paradigm for multi-unit recordings. Neural responses to gap stimuli were analyzed for 212 units (60%) from control mice and 262 units (67%) from prestin-null mice. The distribution of minimal gap thresholds (**Figure 4C**) was shifted leftward, indicating shorter MGTs, in wild-type mice compared to prestin KO controls. We divided our analysis of MGTSs depending on the unit’s temporal response pattern to the noise carrier. Two primary type of temporal response patterns are observed in the IC, phasic or tonic unit types (**Figure 4D**). Phasic units respond to the onset of a noise burst and decay quickly to <5% of the maximum response while tonic units maintain a sustained response throughout the duration of the noise burst. Tonic units showed no dependency to stimulus level (80, 70, or 60 dB) and we observed no significant difference between prestin KO and controls at 80 dB. Phasic units, however, showed a trend of increasing MGT with decreasing stimulus level in the wild-type mice (80 dB: 5.67 ms; 60 dB: 9.95 ms; *p* < 0.05). Additionally, phasic units from wild-type mice had a significantly shorter MGTs compared to phasic units from prestin KO mice at 80-dB presentation level (+/+: 5.67 ms; −/−: 11.46 ms, *p* < 0.001). These results indicate that population gap encoding is driven by phasic units, which show the greatest change across stimulus levels and genotype.

Multiunit gap recovery functions are plotted as NB2 counts with respect to gap duration, which represent the neural correlate of gap detection and again are divided into phasic and tonic units (**Figures 4E,F**). Slopes shown are the mean of all individual unit slopes for each group, WT versus prestin KO. In both tonic and phasic units, prestin KO mice had a decreased slope compared to units from wild-type mice. At the longest gap duration of 96 ms, the NB2 evoked strength of the response (as measured by NB2 counts/repetition) is significantly decreased in the prestin KO mice (phasic: 1.3 counts/rep; tonic: 2.5 counts/rep) compared to wild-type controls (phasic: 2.5 counts/rep; tonic: 3.3 counts/rep). This correlates to the excitatory drive changes seen in the above RLFs and points to diminished excitatory drive in the midbrain of the prestin KO mice.

## Discussion

In this study, we observed that IC neurons from mice with targeted deletion of the prestin gene display a marked decrease in tuning sharpness, excitatory activity, and showed marked increases in pure tone and gap detection thresholds. Specifically, the loss of prestin in cochlear OHCs was linked to broader tuning in the inferior collicular multiunits, decreased excitatory drive as confirmed by tone and noise rate level functions for both NFAEPs and multiunit, a rightward shift in the mean gap thresholds for phasic units, and impairment in gap recovery functions. Taken together, our findings suggest that the impaired tuning and elevated thresholds present in the cochlea due to the loss of the OHC electromotility motor prestin are not compensated for by brainstem and midbrain processing and the loss of prestin loss also impairs midbrain temporal processing.

We did observe a paradoxical decrease in ABR peak 2, 3, and 4 latency in KO mice having moderate to severe SNHL. These peaks originate from brainstem nuclei caudal to the IC including the cochlear nucleus and lateral lemniscus (Land et al., 2017). A plausible explanation is that the weight of contribution of first spike latency of auditory nerve fibers (ANFs) is altered in the prestin KO where broadened receptive fields across the tonotopic axis shift the weight to high frequency (basal) ANFs having shorter latencies. This would effectively decrease the latency of peaks rostral to the auditory nerve. Our failure to observe midbrain compensation with the loss of prestin contrasts with other studies in rats, gerbils, guinea pigs, and humans showing a tight relationship between CAP or cochlear threshold losses and IC or cortical hyperexcitability (Syka, 2002; Kotak et al., 2005; Sun et al., 2009; Thai-Van et al., 2010; Mulders et al., 2011; Lee and Godfrey, 2014; Salvi et al., 2016). In fact, studies of sensorineural hearing loss (SNHL) in the aging C57Bl/6 mouse showed while there was broader IC tuning and fewer high-frequency IC neurons, as we have observed with the loss of prestin, there was an increase in IC hyperactivity and this hyperactivity resulted in an increased acoustic startle reflex (Barsz et al., 2007; Xiong et al., 2017). However, in the aging C57Bl/6 mouse, the loss of thresholds and broader tuning did not result in significant differences in IC neuron mean gap thresholds or in the slopes of the gap recovery function, suggesting that moderate high-frequency SNHL does not affect temporal processing as measured by IC gap detection paradigms (Walton, 2010; Walton et al., 2007).

The prestin KO mouse model of congenital SNHL provides an interesting comparison to study the effects of peripheral hearing impairment on central auditory processing. Unlike other models of acquired hearing loss the prestin KO has moderate to severe SNHL at the onset of hearing so that the effects of homeostatic plasticity maybe reduced (Manzoor et al., 2013). Near-field as well as multi-unit population RLFs are markedly disparate between KO and WT mice showing a threshold shift of ∼55 dB and increased slopes above threshold. Similar results are observed in cats with acoustic trauma with broadly tuned, tuning curves. Receptive fields in all neurons recorded from KO mice are also broadly tuned, regardless of BF, similar to ANF tuning curves measured in animals with moderate SNHL resulting from acoustic trauma (Heinz et al., 2005). A similar finding following acoustic trauma has been reported by Cai et al. (2009) who found that ventral cochlear nucleus neurons with primary-like responses displayed increased RLF slopes.

The temporal processing deficits we observed in the prestin KO mice may be due to the loss of prestin throughout development. In fact, Snell and Frisina (2000) found that in adults, age-related declines in gap detection may begin earlier than age-related changes in word recognition. In fact, Snell et al. (2002) found that when adults with normal audiometric thresholds were compared, those with impaired temporal processing (or gap detection) exhibited impaired speech understanding. We observed prolonged neural MGTs and NB2 recovery functions in sound evoked activity to NB2 to a gap in noise paradigm from neurons in prestin KO mice at low sensation levels. However, mean neural MGTs were comparable between prestin null and wild-type mice when the sensation level of the gap carrier was approximated. Psychoacoustic deficits in hearing impaired human listeners temporal processing following SNHL vary depending on the type of signal for human listeners. Minimal deficits are observed for amplitude modulation and appear to depend on whether the envelope or temporal fine structure are measured (Fitzgibbons and Gordon-Salant, 1987; Nelson and Thomas, 1997; Fullgrabe et al., 2003). Neural correlates of envelope coding are also within normal limits (Kale and Heinz, 2010, 2012). Similarly, Horwitz et al. (2011) reported no significant impairment in MGTs in young listeners with mild to moderate SNHL when sensation level was equated. In the case of moderate SNHL of 40–60 dB threshold elevation as is observed in the prestin KO mice deficits in stimulus onset coding, such as measured with the gap in noise paradigm, regardless of stimulus level can be considered to be a significant impairment (Fitzgibbons and Gordon-Salant, 1987).

As mentioned earlier, our studies were performed on young 1-month-old prestin (−/−) KO mice reported by Liberman et al. (2002), however, in this mouse model, the OHCs have altered mechanical properties. Yet, when a newer mouse model 499KI was generated (Dallos et al., 2008) that did not have altered OHC mechanical properties, the 499KI mouse still exhibited increased cochlear thresholds and broadened cochlear tuning (Weddell et al., 2011). We presume that the 499KI mouse would also have similar midbrain temporal processing deficits and broadened tuning. The broadened tuning we observed from IC midbrain FRAs were only slightly improved from cochlear tuning curves obtained from cochlear CAPs from prestin (−/−) KO or 499KI mice (Cheatham et al., 2004; Weddell et al., 2011).

Cheatham et al. (2009) created many chimeric animals to investigate the effect the effect that different proportions of prestin-containing OHCs had on cochlear amplification. They were able to reduce the number of prestin-containing OHCs in a dose-dependent fashion and found a proportional reduction in sensitivities and tip-lengths of CAP tuning curves as animals contained more of the prestin (−/−) genome. Perhaps, the complete loss of the prestin gene, as in our study, is too overwhelming for significant brainstem and midbrain compensation. It would be interesting to determine if less severe prestin deficits as observed in prestin-chimeras would allow central processing to overcome deficits in midbrain tuning and gap detection. In summary, our findings on young prestin (−/−) KO mice and subsequent deleterious effects on midbrain tuning and temporal processing point the importance of early interventions to treat moderate to severe SNHL.

## Author Contributions

JW, AD, OV, and AL conceived and designed the experiments, and participated in the acquisition, analysis, and interpretation of the experimental findings. JW, AD, and AL wrote and revised the manuscript. AL and JW gave final approval and are accountable to all aspects of the work.

## Conflict of Interest Statement

The authors declare that the research was conducted in the absence of any commercial or financial relationships that could be construed as a potential conflict of interest.
